# Exploring a ferroptosis and oxidative stress-based prognostic model for clear cell renal cell carcinoma

**DOI:** 10.3389/fonc.2023.1131473

**Published:** 2023-03-30

**Authors:** Dongxu Lin, Bintao Hu, Shiqing Zhu, Yue Wu

**Affiliations:** ^1^ Department of Urology, Tongji Hospital, Tongji Medical College, Huazhong University of Science and Technology, Wuhan, Hubei, China; ^2^ Institute of Urology, Tongji Hospital, Tongji Medical College, Huazhong University of Science and Technology, Wuhan, Hubei, China

**Keywords:** clear cell renal cell carcinoma, ferroptosis, oxidative stress, prognostic model, bioinformatics

## Abstract

**Background:**

Ferroptosis is a newly defined cell death process triggered by increased iron load and tremendous lipid reactive oxygen species (ROS). Oxidative stress-related ferroptosis is of great important to the occurrence and progression of clear cell renal cell carcinoma (ccRCC), which is particularly susceptibility to ferroptosis agonist. Therefore, exploring the molecular features of ferroptosis and oxidative stress might guide the clinical treatment and prognosis prediction for ccRCC patients.

**Methods:**

The differentially expressed ferroptosis and oxidative stress-associated genes (FPTOSs) between normal renal and ccRCC tissues were identified based on The Cancer Genome Atlas (TCGA) database, and those with prognostic significances were applied to develop a prognostic model and a risk scoring system (FPTOS_score). The clinical parameter, miRNA regulation, tumor mutation burden (TMB), immune cell infiltration, immunotherapy response, and drug susceptibility between two FPTOS-based risk stratifications were determined.

**Results:**

We have identified 5 prognosis-associated FPTOSs (*ACADSB*, *CDCA3*, *CHAC1*, *MYCN*, and *TFAP2A*), and developed a reliable FPTOS_socre system to distinguish patients into low- and high-risk groups. The findings implied that patients from the high-risk group performed poor prognoses, even after stratified analysis of various clinical parameters. A total of 30 miRNA-FPTOS regulatory pairs were recognized to identify the possible molecular mechanisms. Meanwhile, patients from the high-risk group exhibited higher TMB levels than those from the low-risk groups, and the predominant mutated driver genes were *VHL*, *PBRM1* and *TTN* in both groups. The main infiltrating immune cells of high- and low-risk groups were CD8^+^ T cells and resting mast cells, respectively, and patients from the high-risk groups showed preferable drug responsiveness to anti-PD-1 immunotherapy. Eventually, potential sensitive drugs (cisplatin, BI-D1870, and docetaxel) and their enrichment pathways were identified to guide the treatment of ccRCC patients with high-risk.

**Conclusion:**

Our study comprehensively analyzed the expression profiles of FPTOSs and constructed a scoring system with considerable prognostic value, which would supply novel insights into the personalized treatment strategies and prognostic evaluation of ccRCC patient.

## 1 Introduction

Renal cell carcinoma (RCC) is one of the most common malignant genitourinary tumors. There are 431,288 newly diagnosed cases and 179,368 newly dead cases worldwide in 2020 (1), and it is estimated that there are 81,800 new cases and 14,890 dead cases in the United States in 2023 (2). The incidence of RCC continued increasing at a rate of approximately 1% annually, while mortality rates have decreased by about 2% annually from 2016 to 2020, which might be attributed to advancements in diagnostic tools and early treatment (2). Clear cell renal cell carcinoma (ccRCC) represents the predominant pathological subtype, accounting for almost 70% of all RCC (3). Although 70% of early localized RCC tumor can be completely surgery resection by radical nephrectomy, there is still up to 30% of patients will eventually progress to distant metastasis (3, 4). The ccRCC patients with advanced stage are likely to experience poor outcomes, and the 5-year overall survival (OS) rate is only 11.7% (5). Despite there are occasional reports of durable responses, most advanced RCC patients will develop resistance to targeted drugs such as first-line VEGFR inhibitor (sunitinib, pazopanib) and second-line mTOR inhibitor (everolimus) (6, 7). Therefore, seeking for molecular biomarkers with accurate predictive capacity and therapeutical potential has attract the concerns of many scholars.

Crosstalk between ferroptosis and oxidative stress has been demonstrated in many diseases, such as ischemic stroke (8), inflammation (9), and cancer (10). Ferroptosis is a newly defined nonapoptotic programmed cell death type, characterized by active iron overload, excessive lipid reactive oxygen species (ROS) generation and membrane phospholipid peroxidation (11). In brief, when the redox homeostasis is impaired, iron generates active hydroxyl radical (·OH) *via* Fenton reaction, which then promotes the production of phospholipid hydroperoxides (PLOOH). Meanwhile, blocking of cystine/glutamate antiporter system Xc^-^ decreases the synthesis of glutathione (GSH) and the only intracellular PLOOH-neutralizing enzyme glutathione peroxidase 4 (GPX4), and eventually contributes to the accumulation of ROS and ferroptosis (12). Oxidative stress is occurred due to the breakdown of the redox homeostasis, characterized by an increase of ROS and a decrease of antioxidant enzymes (13). ROS at physiological level is essential to maintain the function of cellular biology, however, excessive ROS generation under oxidative stress condition is a double-edged sword for cancer (14). For one thing, ROS-caused oxidative damage promotes cell death (apoptosis, ferroptosis) and triggers anti-tumor immune cells (M1 macrophages, T cells) infiltration to function as a tumor suppressor (15). Besides, high level of ROS causes detrimental damages of DNA, protein, and lipid, and induces genomic instability to function as a tumor promoter (16). In general, exacerbating ROS generation and undermining antioxidant system are sufficient to trigger oxidative stress and ferroptosis in tumor cells (17).

Sensitivity analysis of ferroptosis agonist erastin on 177 cancer cell lines indicated that RCC and diffuse large B cell lymphoma were extremely susceptible to GPX4-dependent ferroptosis (18). Hence, targeting ferroptosis and oxidative stress may challenge the current treatment paradigm of RCC. Previous studies usually consider the impact of a single gene or variable on the ccRCC development. However, a widely accepted consensus is that tumorigenesis and progression were affected by the interaction of multiple factors in a sequential and coordinated manner. Thus, it is urgent to develop an integrative and efficient utility to reflect the features of ferroptosis and oxidative stress in ccRCC. With the advances in multiomic sequencing, it is possible to comprehensively explore the genomic profiles of ccRCC. Here, we had identified differentially expressed ferroptosis and oxidative stress-associated genes (FPTOSs), and 5 genes with independent prognostic values were incorporated into the prognostic model. Subsequently, all ccRCC patients were allocated into low- and high-risk groups according to the FPTOS_score, and the prognostic significance of FPTOS-based risk stratification was assessed in both the TCGA-KIRC and E-MTAB-1980 cohorts. The miRNA regulation, mutation pattern, immune cell population, immunotherapy responsiveness, and drug susceptibility were also examined.

## 2 Materials and methods

### 2.1 Data collection and preprocessing

Transcriptome data, clinical parameters and prognosis data, miRNA sequencing data, and somatic mutation data of ccRCC patients were extracted from The Cancer Genome Atlas (TCGA) database (https://portal.gdc.cancer.gov/). E-MTAB-1980 cohort was acquired from ArrayExpress database (https://www.ebi.ac.uk/arrayexpress/) and served as the external validation dataset. The raw data from TCGA-KIRC cohort were preprocessed through averaging the expression levels of same genes, removing the genes with low expression levels below 1, and normalizing the expression profiles using trimmed mean of M-values (TMM) method based on the edgeR package. As for the microarray data from E-MTAB-1980 cohort, we performed background adjustment and normalization using the robust multiarray analysis (RMA) method based on Affy package. Furthermore, the expression values were log2 transformed, and the probes were converted into corresponding gene symbols.

### 2.2 Preparation of ferroptosis and oxidative stress-associated gene set

Ferroptosis-associated genes were gained from the FerrDb database (http://www.zhounan.org/ferrdb/current/). To obtain oxidative stress-associated genes, we applied “oxidative stress” as search term to acquire genes that were involved in the process of oxidative stress from the OMIN database (https://www.oncomine.org/resource/), NCBI gene function module (https://www.ncbi.nlm.nih.gov/gene/) and GeneCard database (https://www.genecards.org/). We then acquired the integrative gene set from the TCGA-KIRC cohort. After that, ferroptosis and oxidative stress-associated gene set was prepared by selecting the intersecting genes among above gene sets using Venn diagram.

### 2.3 Development and validation of a FPTOS-based prognostic model

Differentially expressed FPTOSs of ccRCC patients were identified through R package “EdgeR” referring to screening criteria of |log2 fold change (FC)| > 1 and adjusted *P* < 0.05. Subsequently, univariate Cox regression, least absolute shrinkage and selection operator (LASSO) regression, and multivariate Cox regression analyses were utilized to investigate the FPTOSs with prognostic significance of ccRCC. The individualized risk score of each ccRCC patient, named FPTOS_score, was measured using the formula: $FPTOS_{-}score=\sum_{i=1}^{n}Expi\beta i$ . Of that, *Exp* denoted the expression level of specific gene, while *β* represented the corresponding regression coefficient. On basis of the median value of FPTOS_score, all ccRCC patients were allocated into low- and high-risk groups. Subsequently, Kaplan-Meier method was used to explore the prognosis difference between two risk groups, and receiver operating characteristic (ROC) curve was plotted to estimate the power and accuracy of FPTOS-based prognostic model. The external validation cohort (E-MTAB-1980) was applied to assess the predictive performance and stability of the prognostic model. Meanwhile, the prognostic values of the FPTOSs were verified separately based on the GEPIA database (http://gepia.cancer-pku.cn/index.html).

We first compared the difference in the number of deaths between two risk stratifications, and calculated the FPTOS_score of alive and dead patients, so as to reveal whether FPTOS-based risk stratification could distinguish patients with poor prognosis. In order to discover independent prognostic factors of ccRCC, FPTOS_score and various clinical parameters including age, gender, grade, stage, T stage, N stage, M stage were subjected to univariate and multivariate Cox regression analyses. Furthermore, stratified analyses of various clinical parameters were conducted to determine whether FPTOS-based risk stratification still performed a considerable prognostic value.

### 2.4 Construction of miRNA-FPTOS regulatory network

miRNA sequencing data were extracted from TCGA-KIRC cohort, and the differentially expressed miRNAs were determined *via* comparing the expression differences between the normal and tumor samples with the setting criteria of |log2 FC| > 1 and *P* < 0.05. Then we investigated the co-expression patterns between miRNAs and prognostic-associated FPTOSs, and mapped miRNA-FPTOS regulatory pairs on the basis of filtering criteria (|cor| > 0.25, *P* < 0.001).

### 2.5 Tumor mutation burden (TMB) analysis

R package “Maftool” was applied to determine the TMB levels using somatic mutation data from the TCGA database. Survival analysis was applied to determine the influence of TMB on the outcome of ccRCC patients. The TMB levels in two risk stratifications and their correlations with FPTOS_score were also measured. TMB was estimated *via* counting the overall number of mutations per coding in the tumor sample. Moreover, waterfall diagrams were plotted to display the landscape of gene mutation profiles in two risk stratifications. We then evaluated the predictive capacities of risk stratification on the ccRCC patients’ prognosis when the mutation of driver genes such as *VHL*, *PBRM1* and *TNN* were considered.

### 2.6 Exploration of immune microenvironment and response to immunotherapy

The abundances of immune cell types between two risk stratifications was evaluated by the CIBERSORT approach and LM22 signature matrix (19). We performed 1000 permutation tests to ensure the stability of the outputs. The immune microenvironment was investigated using ESTIMATE algorithm according to the predictive results of immune score, estimate score and tumor purity (20).

In order to determine the immunotherapy responsiveness, we subsequent analyzed the expression profiles of immune checkpoint inhibitor (ICI)-targeted genes (*PD-1*, *CTLA-4*) between two risk stratifications. Taken the mutation profiles of ICI-targeted genes into account, the influence of FPTOS_score on the patients’ prognosis was explored. Since the lack of available ccRCC cohorts receiving immunotherapy, we employed the Tumor Immune Dysfunction and Exclusion (TIDE) algorithm to predict the responsiveness towards immunotherapy (21). Applying an open-access immunotherapy-treated melanoma cohorts, unsupervised subclass mapping (SubMap) method was utilized to indirectly predict the immunotherapy responsiveness in the two risk stratifications according to the similarity of gene expression profile (22). Additionally, adopting expression and survival data from a metastatic melanoma cohort who receiving PD-1 immunotherapy, we further conducted survival analysis to evaluate the progression-free survival (PFS) rates of different risk groups.

### 2.7 Identification of sensitive drugs based on FPTOS_score

The transcriptional data, drug susceptibility data, and corresponding drug targets or pathways of various tumor cell lines were extracted from a pharmacogenomic dataset Genomics of Drug Sensitivity in Cancer (GDSC, https://www.cancerrxgene.org/). The relationship between the drug susceptibility and the FPTOS_score was evaluated by Person correlation analysis according to the criteria (|correlation coefficient (R) | > 0.15 and *P* < 0.05). The targets or pathways of these drugs were also screened out to estimate the underlying mechanisms.

### 2.8 Real-time PCR (RT-PCR) analysis

To examine the expression level of the identified FPTOSs in ccRCC sample, we further carried out RT-PCR experiments to compare the mRNA expression difference between human ccRCC tumor specimen and adjacent normal specimen. Moreover, the mRNA expression of FPTOSs in human normal renal proximal tubular cell line (HK2), human renal clear cell carcinoma cell lines (786-O, OS-RC-2) were also evaluated. Cells was purchased from Shanghai Cell Bank Type Culture Collection Committee (Shanghai, China) and incubated in RPMI-1640 medium containing 10% fetal bovine serum (FBS). The total RNA was extracted using Trizol reagent and then transcribed into cDNA using 1st Strand cDNA Synthesis Kit (Vazyme, China). RT-PCR method was performed *via* qPCR SYBR Green Master Mix (Vazyme, China) in a QuantStudio™ 6 Flex Real-Time PCR System. The result was normalized to housekeeping gene GAPDH, and the selected primers for the FPTOSs were listed in **Table S1**.

### 2.9 Statistical analysis

The statistical analysis and result presentation were realized *via* R version 4.0.5 and GraphPad Prism version 8.0. Unpaired student’s *t* test or Mann-Whitney *U* test was utilized to investigate the differences between two groups with or without normally distributed variables, respectively. Log-rank test was applied to compare different survival outcomes between two groups. Correlation analysis between two continuous variables was realized by either Pearson or Spearman test as appropriate. Contingency table variables were processed with Chi-squared (χ^2^) test or Fisher’s exact test. Unless otherwise stated, *P* < 0.05 was regarded as statistically significant for all analysis.

## 3 Results

### 3.1 Identification of FPTOS gene signature

**Figure 1:**
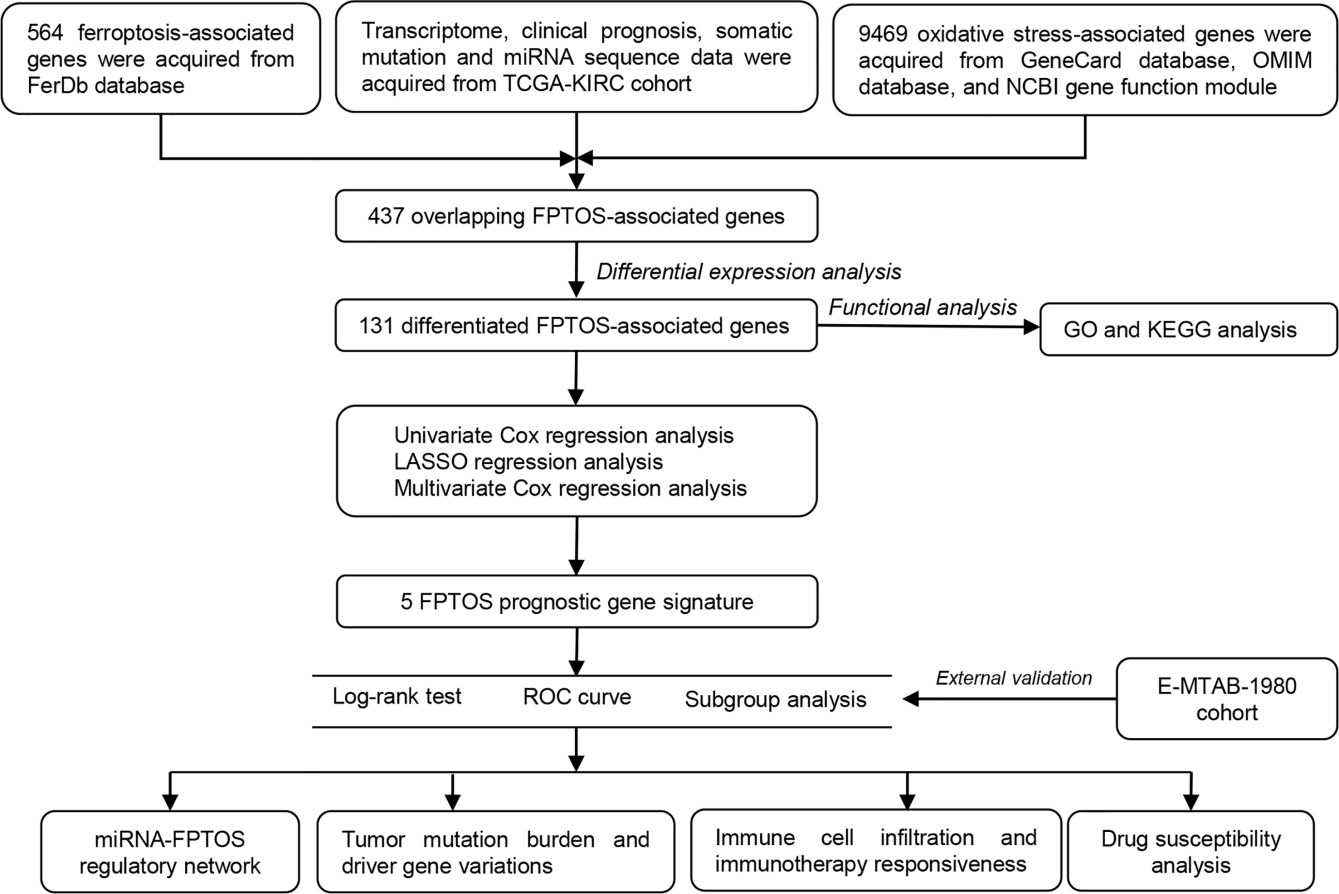
Flowchart depicts the searching procedures to develop a FPTOS-based prognostic model in ccRCC.

**Figure 2:**
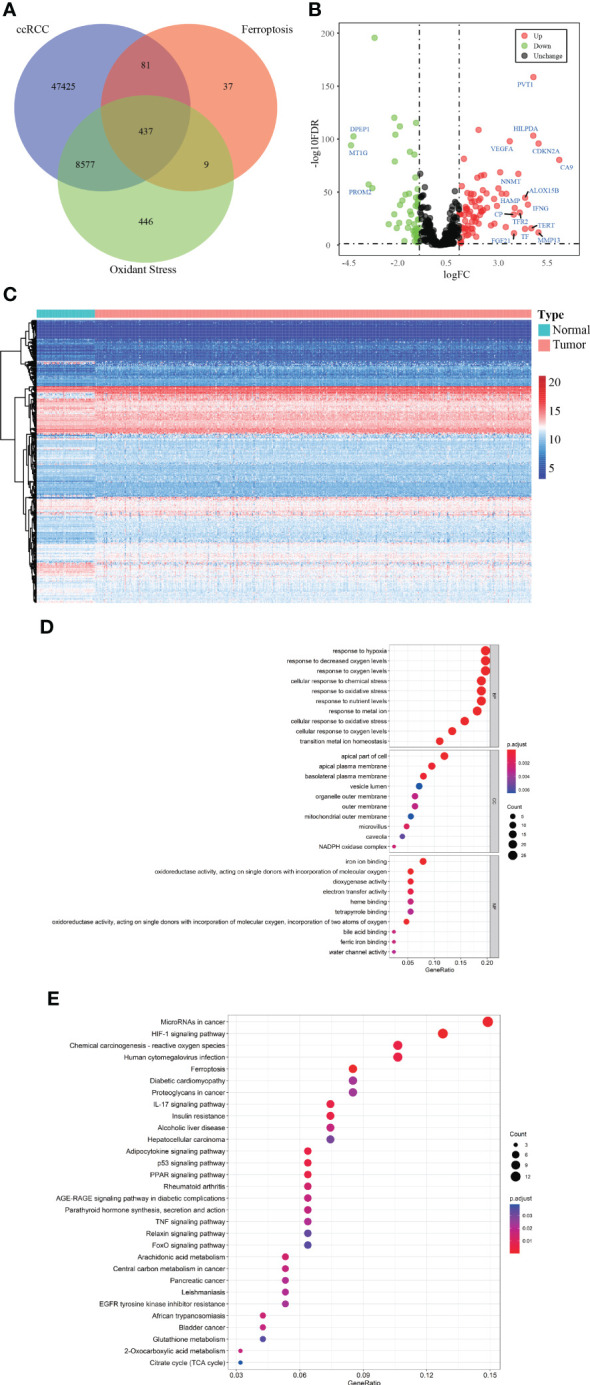
Identification of the differentially expressed FPTOSs of ccRCC in TCGA database. **(A)** Searching for FPTOS-associated genes in ccRCC patients using Venn diagram. **(B)** Visualization of differentially expressed FPTOSs between normal renal tissues (N = 72) and ccRCC tissues (N = 539) using volcano plot based on the transcriptional data in TCGA-KIRC cohort. **(C)** Visualization of differentially expressed FPTOSs using heatmap based on transcriptional data in TCGA-KIRC cohort. **(D)** GO enrichment analysis of differentially expressed FPTOSs to determine involved gene function. **(E)** KEGG enrichment analysis of differentially expressed FPTOSs to determine involved pathway.

**Figure 1** depicted the selection procedures of FPTOS-based prognostic signature. Specifically, we first obtained transcriptome data of ccRCC patients from the TCGA-KIRC cohort, which included 72 normal renal specimens and 539 ccRCC tumor specimens. A Venn diagram was plotted to identify all genes of interest that was closely associated with ferroptosis and oxidative stress, and a total of 437 FPTOSs were output for further analysis (**Figure 2A**). Subsequently, the differentially expressed FPTOSs between normal and tumor specimens were screened out based on the filtering criteria (|log2 FC| > 1.0, *P* < 0.05), and 50 downregulated genes and 81 upregulated genes met the requirement. The expression and distribution profiles of these FPTOSs were presented in **Figures 2B, C**.

We then carried out GO and KEGG enrichment analyses to determine the biological functions and involved pathways of the FPTOSs. The biological processes were enriched in the responses to hypoxia, oxygen levels, chemical stress and oxidative stress. The cell components lied in apical part of cell, apical plasma membrane, and basolateral plasma membrane. With regard to molecular functions, these genes were involved in iron ion binding, oxidoreductase activity, acting on single donors with incorporation of molecular oxygen, and dioxygenase activity (**Figure 2D**). Additionally, KEGG analysis indicated that the identified genes were related with miRNAs in cancer, HIF-1 signaling pathway, carcinogenesis-reactive oxygen species, human cytomegalovirus infection, and ferroptosis (**Figure 2E**). The findings revealed that the differentially expressed FPTOSs were primarily implicated in hypoxia, oxidative stress, ferroptosis and oxygen level regulation, confirming that the filtering criteria could accurately recognize the FPTOSs of interest.

### 3.2 Development and validation of a FPTOS-based prognostic model

**Figure 3:**
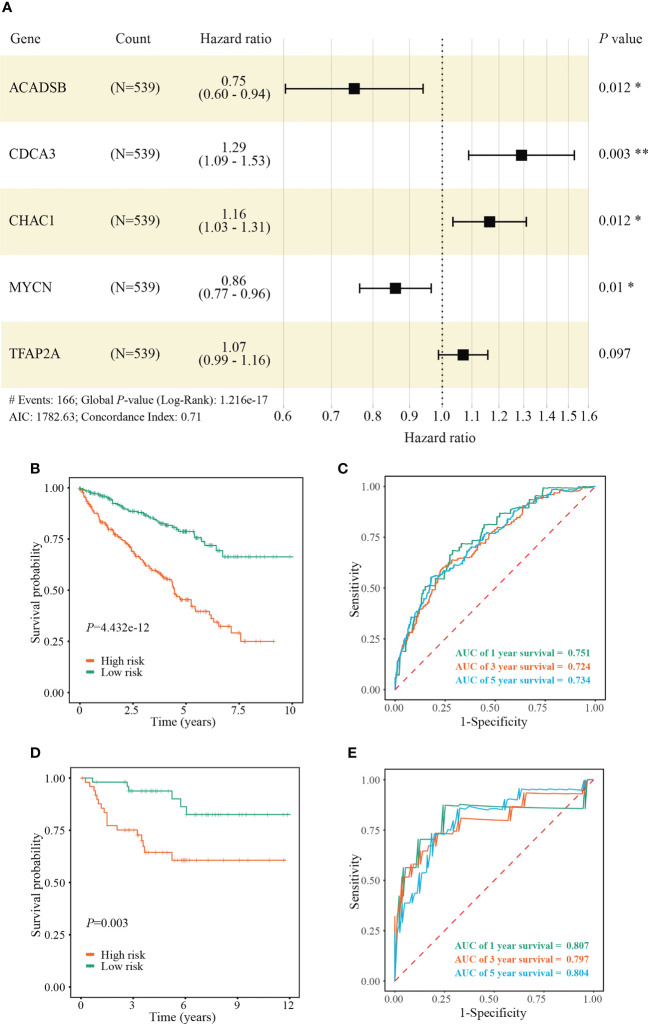
Construction and validation of a FPTOS-based prognostic model. **(A)** Multivariate Cox regression analysis to evaluate the prognostic values of 5 FPTOSs. **(B)** Kaplan-Meier survival curve analysis to compare overall survival (OS) difference between low- and high-risk groups in the TCGA-KIRC cohort. **(C)** Time-dependent ROC curve analysis to evaluate the predictive power of the FPTOS-based risk stratification in the TCGA-KIRC cohort. **(D)** Kaplan-Meier survival curve analysis to compare OS difference between low and high-risk groups in the validated E-MTAB-1980 cohort. **(E)** Time-dependent ROC curve analysis to evaluate the predictive power of the FPTOS-based risk stratification in the validated E-MTAB-1980 cohort. Log-rank test was applied to compare the statistical differences in the Kaplan-Meier curves.

We identified 131 FPTOS-related prognostic genes by univariate Cox regression analysis (**Table S2**). LASSO regression analysis was carried out to search the predominant prognostic FPTOSs. The trajectory variations in regression coefficients of above 131 genes were presented in **Figure S1A**, and the cross-validation results of LASSO model construction were presented in **Figures S1B**. Finally, 6 output genes (*ACADSB*, *BID*, *CDCA3*, *CHAC1*, *MYCN* and *TFAP2A*) were identified and subjected for further study. Applying multivariate Cox regression analysis, 5 genes (*ACADSB*, *CDCA3*, *CHAC1*, *MYCN*, *TFAP2A*) with independent prognostic significances were incorporated into the prognostic model (**Table 1**; **Figure 3A**). Among them, *ACADSB* and *MYCN* were considered as the protective factors, while *CDCA3*, *CHAC1*, and *TFAP2A* were considered as the detrimental factors. Furthermore, we examined the prognostic values of the identified FPTOSs in the ccRCC patients. Based on the expression profiles and outcome data in the GEPIA database, we found that *ACADSB* and *MYCN* are the favorable prognostic marker of ccRCC, while *CDCA3*, *CHAC1*, and *TFAP2A* are the unfavorable prognostic marker of ccRCC (**Figure S2**). The above findings further highlighted the considerable prognostic capacities of the FPTOSs in monitoring ccRCC progression.

**Table 1:** Multivariate Cox regression analysis to identify prognosis-related FPTOSs.

Gene	Coef	Exp (coef)	se (coef)	z	Pr (>|z|)
*ACADSB*	-0.2832	0.7534	0.1130	-2.5057	0.0122
*CDCA3*	0.2549	1.2904	0.0868	2.9370	0.0033
*CHAC1*	0.1523	1.1645	0.0603	2.5261	0.0115
*MYCN*	-0.1508	0.8600	0.0587	-2.5688	0.0102
*TFAP2A*	0.0672	1.0695	0.0405	1.6575	0.0974

Coef, coefficient.

The FPTOS_score of each ccRCC patient was computed applying the following formula: FPTOS_score = (-0.2832 × Exp *ACADSB*) + (0.2549 × Exp *CDCA3*) + (0.1523 × Exp *CHAC1*) + (-0.1508 × Exp *MYCN*) + (0.0672 × Exp *TFAP2A*). To assess the model applicability, the ccRCC patients were allocated into the low- and high-risk groups on the basis of the median value of FPTOS_score. The difference of OS between two risk stratifications from the TCGA-KIRC cohorts was measured by Kaplan-Meier method, and the results suggested that patients from the high-risk group performed a worse prognosis than those from the low-risk group (*P* = 4.432e-12, **Figure 3B**). The ROC curve was also plotted to evaluate the prediction power and accuracy of FPTOS-based risk stratification. As presented in **Figure 3C**, the area under the ROC curve (AUC) values were 0.751 at 1-year, 0.724 at 3-year, and 0.734 at 5-year. Furthermore, external validation was applied to evaluate whether the prognostic model showed stable performance in the E-MTAB-1980 cohort. As a result, a poor prognosis was observed in the high-risk group (*P* = 0.003, **Figure 3D**), and the AUC values of 1-year, 3-year, and 5-year OS rates were 0.807, 0.797, and 0.804 (**Figure 3E**). Generally, these findings indicated a preferable predictive power and stability of the FPTOS-based prognostic model.

### 3.3 Independence of the FPTOS_score from clinical parameters of ccRCC

**Figure 4:**
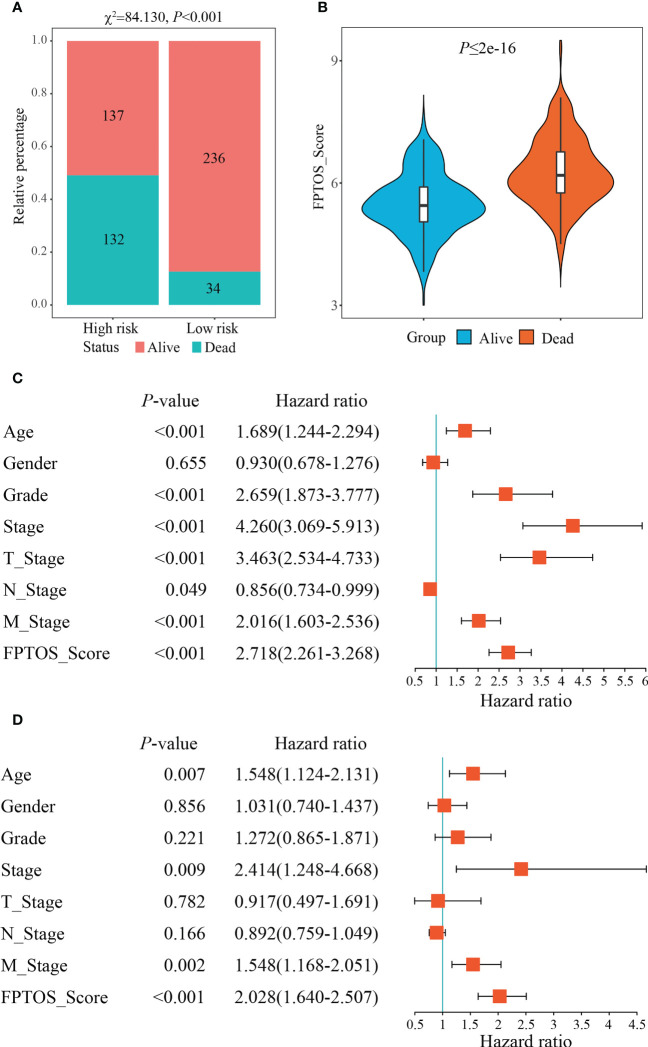
Independence of the FPTOS_score from clinical parameters of ccRCC. **(A)** Survival status of low- and high-risk groups stratified by FPTOS_score in ccRCC patients. The categorical variables were analyzed with the Chi-squared (χ^2^) test. **(B)** FPTOS_score of ccRCC patients stratified by survival status. **(C, D)** Univariate or multivariate Cox regression analysis to confirm the independent prognostic significance of FPTOS_score and clinical parameters for ccRCC patients.

We then investigated the survival outcomes between two FPTOS-based risk stratifications, and it is shown that ccRCC patients with high-risk exhibited lower OS rates than those with low-risk (χ^2^= 84.130, *P* < 0.001) (**Figure 4A**). Similarly, the dead patients performed a higher FPTOS_score than the alive patients (*P* ≤ 2e-16) (**Figure 4B**), indicating a positive correlation between FPTOS_score and poor prognosis. To further confirm the independence of FPTOS_score on the prognostic evaluation of ccRCC, the crucial clinical parameters (age, gender, grade, stage, T stage, N stage, M stage) and FPTOS_score were subjected to univariate and multivariate Cox regression analyses (**Table S3**; **Figures 4C, D**). The findings suggested that FPTOS_score could serve as an independent prognostic variable of ccRCC patients (HR = 2.028, 95% CI: 1.640-2.507, *P* < 0.001).

We next investigated the feasibility of the FPTOS-based risk stratification in predicting the prognosis of ccRCC patient subgroups stratified by above clinical parameters. As the results acquired from the Kaplan-Meier survival analyses, the survival prognosis of ccRCC patients with high-risk were significantly worse than those with low-risk, regardless of the clinical variable stratifications (All *P* < 0.001) (**Figures S3A–S3N**). Such results implied that FPTOS-based risk stratification could distinguish patients with poor outcomes without considering the influence of other clinical parameters.

### 3.4 Construction of miRNA-FPTOS regulatory network

**Figure 5:**
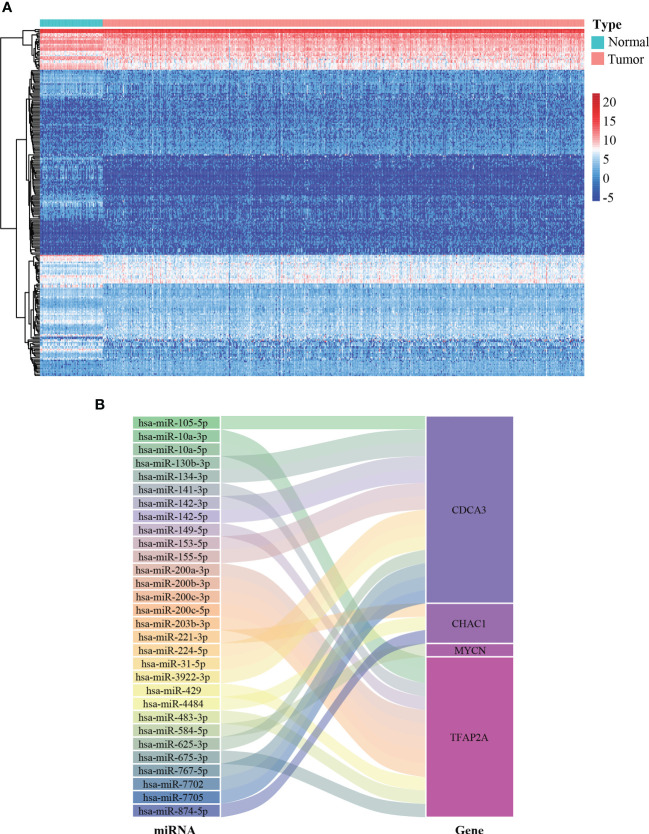
Construction of miRNA-FPTOS regulatory network for ccRCC patients. **(A)** Heatmap of differentially expressed miRNAs between normal renal samples and ccRCC tumor samples. **(B)** Sankey plot to visualize the potential regulatory relationship between differentially expressed miRNAs and prognostic FPTOSs.

miRNAs are implicated in multiple cellular processes including redox homeostasis regulation (23). Therefore, it is valuable to map the miRNA-FPTOS regulatory network, which may underlie the upstream regulatory mechanism of FPTOSs. We first extracted the miRNA sequencing data from the TCGA database. Abnormally expressed miRNAs were identified according to filtering criteria (|log2 FC| > 1.0, *P* < 0.05), and were displayed in heatmap (**Figure 5A**). Then the co-expression analysis between prognostic FPTOSs and abnormally expressed miRNAs was conducted in reference to the inclusion criteria (|cor| > 0.25, *P* < 0.001). A total of 30 miRNA-FPTOS regulatory pairs were screened out (**Table S4**), and a Sankey diagram was plotted to exhibit the regulatory network (**Figure 5B**).

### 3.5 Association between FPTOS_score and mutation profiles

**Figure 6:**
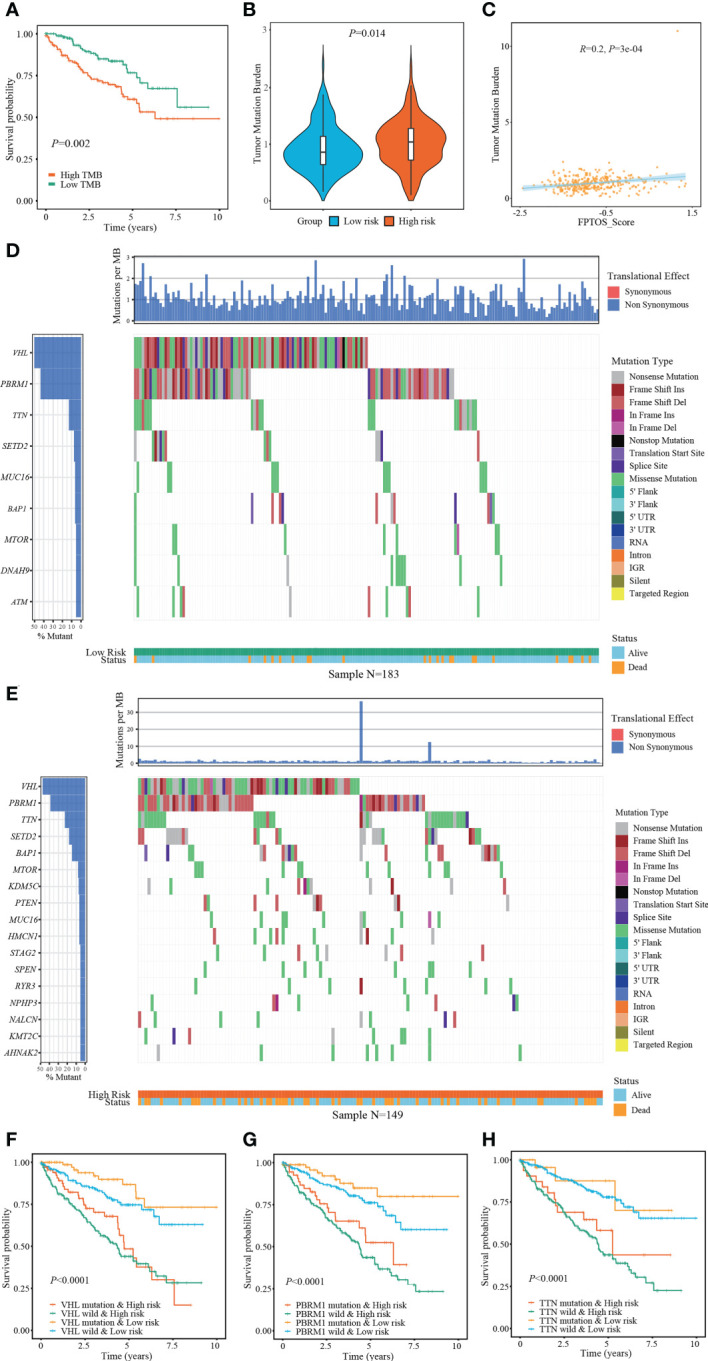
Exploring association between FPTOS_score and mutation profiles. **(A)** Kaplan-Meier survival analysis to explore the influence of TMB levels on the prognosis of ccRCC patients. **(B)** Differences of TMB levels between the two FPTOS-based risk stratifications. **(C)** Person’s correlation analysis between FPTOS_score and TMB level. **(D, E)** Waterfall plot to exhibit the mutation landscape in the low- or high-risk group, respectively. The high-frequency mutated genes and events were illustrated. **(F–H)** Kaplan-Meier survival analysis among four groups stratified by the FPTOS-based risk stratifications and mutation profiles of driver genes *VHL*, *PBRM1*, or *TTN*, respectively.

The occurrence and progression of ccRCC were partially attributed to the mutation of driver genes. At present, we extracted the somatic mutation data of ccRCC patients from TCGA-KIRC cohort to reveal the association between FPTOS_score and mutation profiles. We found that patients with high TMB levels experienced worse outcomes than patients with low levels (*P* = 0.002) (**Figure 6A**), and elevated TMB levels were observed in the patients from high-risk group (**Figure 6B**). Moreover, correlation analysis suggested that FPTOS_score was positively correlated with TMB level (*R* = 0.20, *P* = 3e-4) (**Figure 6C**).

Subsequently, the genes mutated in at least 5% of the tumor specimens from two risk stratifications were illustrated *via* waterfall plot. A significant abundant mutation events was existed in the specimens from high-risk group, accompanying by an increased dead population (**Figures 6D, E**). We employed the top 3 mutated driver genes (*VHL*, *PRBM1*, *TNN*) to investigate whether the FPTOS_score still had prognostic value when the driver gene mutations were taken into account. The results revealed that *VHL*-mutated patients with low-risk performed significant survival advantages than those with high-risk, meanwhile, *VHL*-wild patients with low-risk also performed significant survival advantages than those with high-risk (**Figure 6F**). Consistent with the performance of different *VHL* phenotype groups, patients with low-risk still experienced better outcomes than those with high-risk, no matter whether the mutation of *PRBM1* and *TNN* occurred (**Figures 6G, H**). Collectively, these findings implied that FPTOS-based risk stratification was positively correlated with TMB level and gene mutation frequency, and patients with relatively low FPTOS_score exhibited favorable prognosis even when the mutation of driver genes were considered.

### 3.6 Determination of immune cell infiltration and immune microenvironment

**Figure 7:**
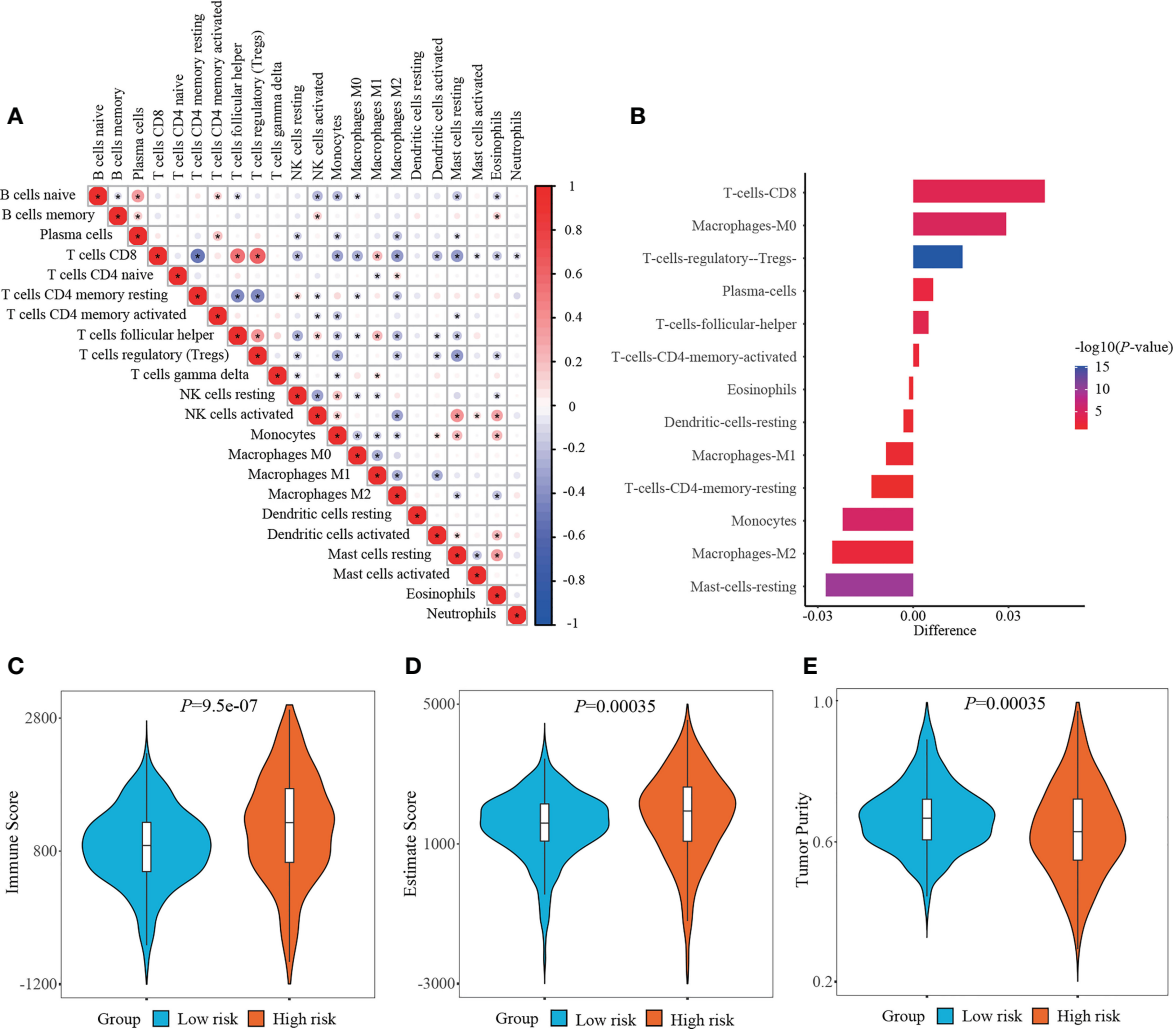
Determining immune cell infiltration and immune microenvironment between two risk stratifications. **(A)** Correlation matrix of all 22 immune cell populations. **(B)** Exploring the differentially infiltration of immune cell populations between low- and high-risk groups. **(C–E)** Comparison of immune score, estimate score or tumor purity using ESTIMATE algorithm between two risk stratifications.

RCC is recently regarded as an immunogenic tumor, which is partly caused by the immune dysfunction with the infiltration of suppressive immune cell subtypes such as regulatory T cells (Tregs) and myeloid-derived suppressor cells (MDSCs) (24). Currently, the components of immune cells were measured using CIBERSORT method. Correlation matrix was plotted to depict all the 22 immune cell proportions, and a strong relevance was existed between CD8^+^ T cells and Tregs in the TCGA-KIRC cohort (**Figure 7A**). It was shown that abundant populations of CD8^+^ T cells, M0 macrophages, and Tregs existed in the patient specimens from high-risk group, while predominant populations of resting mast cells, M2 macrophages, and monocytes accumulated in the specimens from low-risk group (**Figure 7B**).

What else, the immune microenvironment properties of ccRCC specimens were quantified, and the output values of immune score and estimate score in the high-risk group (1152.85 ± 793.65, 1796.53 ± 1239.89, respectively) were significantly higher than those in the low-risk group (860.65 ± 565.16, 1504.52 ± 943.08, respectively), while the output values of tumor purity in the high-risk group (0.6348 ± 0.1311) were significantly lower than those in the low-risk group (0.6712 ± 0.0956) (**Figures 7C–E**).

### 3.7 Evaluation of immunotherapy responsiveness based on FPTOS risk stratification

**Figure 8:**
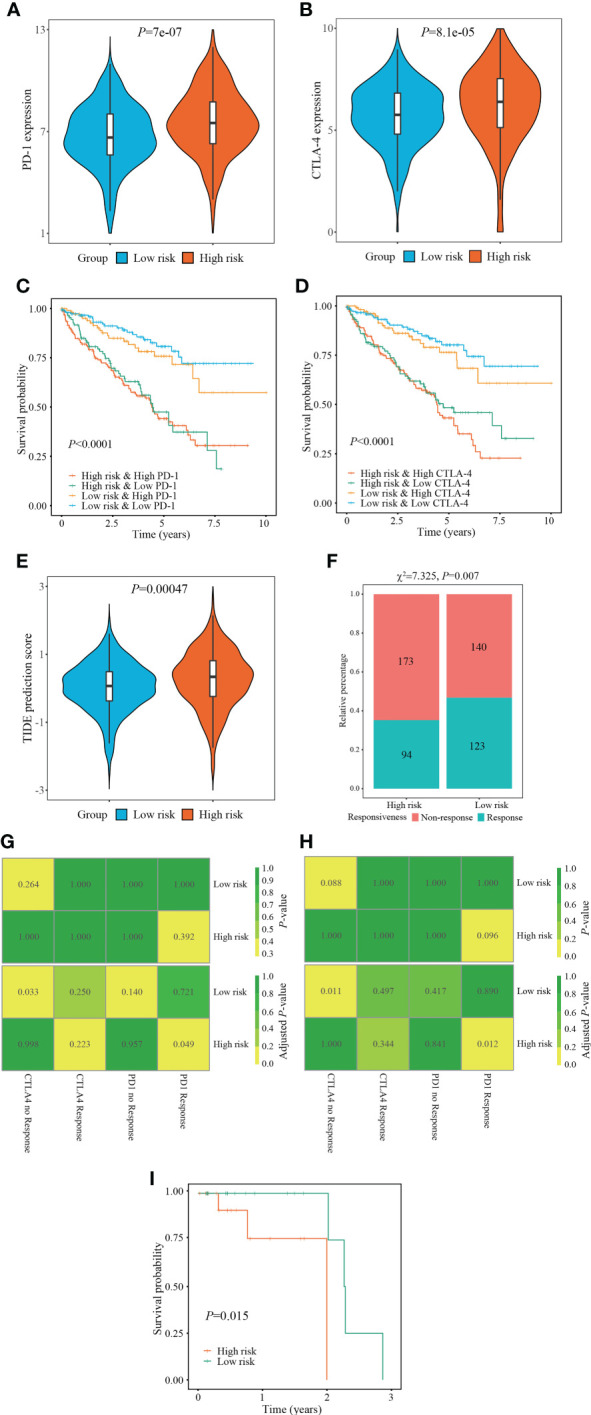
Evaluation of immunotherapy responsiveness based on FPTOS risk stratification. **(A, B)** Expression patterns of ICI targeted gene PD-1 or CTLA-4 in two FPTOS-based risk stratifications. **(C, D)** Kaplan-Meier survival analysis for OS among four groups stratified by the FPTOS-based risk stratifications and PD-1 or CTLA-4 expression level, respectively. **(E)** Difference of TIDE prediction score between the low- and high-risk groups. **(F)** Comparison of immunotherapy responsiveness between low- and high-risk groups. **(G, H)** SubMap analysis to predict the immunotherapy responsiveness in the low- and high-risk groups from the TCGA cohort or GEO cohort, respectively, according to the anti-PD-1 or anti-CTLA-4 responsiveness from the open-access metastatic melanoma cohort. **(I)** Kaplan-Meier survival analysis of progression-free survival (PFS) between the low- and high risk groups in the Riaz’s cohort who have received anti-PD-1 immunotherapy.

Immunotherapy, especially immune checkpoint inhibitor (ICI), has witnessed a tremendous development and revolutionized the treatment of various tumors (25). Therefore, we next measured the changes of ICI targeted genes (*PD-1, CTLA-4*) in different risk stratifications. Compared with the low-risk patients, the expression of *PD-1* and *CTLA-4* in the high-risk patients were dramatically upregulated (all *P* < 0.001) (**Figures 8A, B**). Subsequently, we measured the survival prognosis of ccRCC patients between two risk stratifications when the expression of ICI-targeted genes was taken into consideration. As a result, patients with high-risk and high *PD-1/CTLA-4* expression experienced worse outcomes when compared with patients with low risk and high *PD-1/CTLA-4* level, and patients with high-risk and low *PD-1/CTLA-4* level experienced worse outcomes when compared with patients with low-risk and low *PD-1/CTLA-4* level (**Figures 8C, D**).

Since the absence of easily-accessible ccRCC cohort treated with immunotherapy, the TIDE algorithm, which integrated T cell dysfunction and exclusion on the basis of the expression profiles, was applied to predict the response to immunotherapy. When compared with the low-risk group, the high-risk group presented significantly elevated TIDE prediction scores (*P* = 0.00047) (**Figure 8E**). Meanwhile, patients in different risk stratifications exhibited different immunotherapy responsiveness, while the response ratio of high-risk to low risk was 46.77% to 35.21% (χ^2^ = 7.325, *P* = 0.007) (**Figure 8F**).

Subsequently, the SubMap analysis was conducted to compare the expression characteristics of FPTOS_score acquired from the TCGA and GEO databases with an open-access metastatic melanoma cohort who receiving anti-PD-1 or anti-CTLA-4 treatment. The results revealed that patients with high-risk might respond positively to anti-PD-1 immunotherapy in both TCGA and GEO cohorts (adjusted *P* = 0.049 and 0.012, respectively), conversely, patients with low-risk might respond poorly to anti-CTLA-4 immunotherapy (adjusted *P* = 0.0033 and 0.011, respectively) (**Figures 8G, H**). Furthermore, we evaluated the predictive efficacy of FPTOS_score in the Riaz’s cohort who receiving anti-PD-1 immunotherapy, and discovered that patients with high-risk experienced worse outcomes in PFS when compared with those with low-risk (*P* = 0.015) (**Figure 8I**). These results had provided guidance for the immunotherapy strategy of ccRCC patients, for instance, a feasibility of anti-PD-1 treatment for high-risk patients.

### 3.8 Relationship between FPTOS_score and drug susceptibility

**Figure 9:**
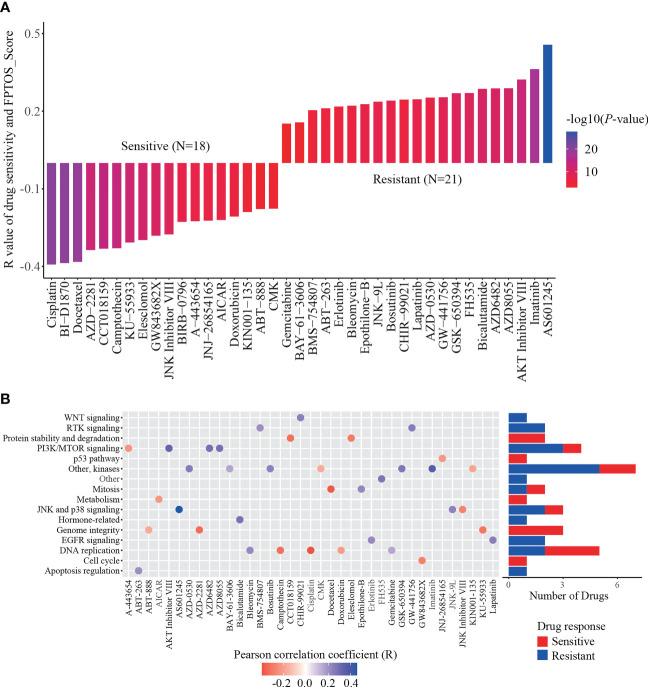
Identification of sensitive drugs for ccRCC patients based on FPTOS_score. **(A)** Person correlation analysis between FPTOS_score and drug susceptibility in the GDSC database. **(B)** Screening for involved pathways of identified drugs.

To explore available drugs for high-risk patients, we further investigated the relevance between FPTOS_score and IC50 values of corresponding drugs in the ccRCC cell lines *via* the pharmacogenomics database GDSC. In the light of inclusion criteria (|R| >0.15, P < 0.05), 18 drugs (including cisplatin, BI-D1870 and docetaxel) performed sensitive responses towards high FPTOS_score, while 21 drugs (including AS601245, AKT Inhibitor VIII and AZD8055) performed resistant responses towards high FPTOS_score (**Figure 9A**). What else, the drug-involved pathways were analyzed. As shown in the **Figure 9B**, the sensitive drugs were enriched in the pathways associated with genome integrity, metabolism, p53 pathway, protein stability and degradation, while the resistant drugs were involved in the pathways such as WNT signaling, RTK signaling, hormone-related, EGFR signaling, apoptosis regulation and Other. The above findings indicated that the FPTOS_score might influence the drug responsiveness of ccRCC cell lines, which might provide insights into the cancer treatment.

### 3.9 Exploring the expression pattern of the identified FPTOSs

**Figure 10:**
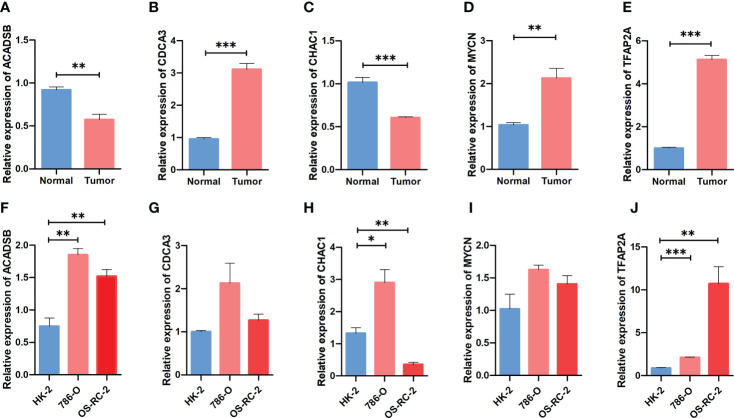
Comparing the expression pattern of the identified FPTOSs between ccRCC and normal renal sample using RT-PCR method. **(A–E)** The mRNA expression level of *ACADSB*, *CDCA3*, *CHAC1*, *MYCN*, *TFAP2A* in human ccRCC tumor samples and adjacent normal samples. **(F–J)** The mRNA expression level of *ACADSB*, *CDCA3*, *CHAC1*, *MYCN*, *TFAP2A* in human ccRCC cell lines (786-O, OS-RC-2) and normal renal proximal tubular cell line (HK2). Results were presented as mean ± standard error of mean (SEM), and *P* < 0.05 was considered to have statistically significant. *P < 0.05, **P < 0.01, ***P < 0.001.

The mRNA expression of prognostic FPTOSs in both renal tissue and cell samples was determined by RT-PCR method. As the results indicated, the expressions of *CDCA3*, *MYCN* and *TFAP2A* in ccRCC tumor tissue were significantly upregulated compared with those in adjacent normal kidney tissue, while the expressions of *ACADSB* and *CHAC1* were significantly downregulated (**Figures 10A–E**). Additionally, the mRNA expression of *ACADSB*, *CHAC1*, and *TFAP2A* were also significantly upregulated in ccRCC cell line 786-O, while the *CHAC1* was downregulated but *ACADSB* and *TFAP2A* were upregulated in another ccRCC cell line OS-RC-2 (**Figures 10F–J**).

## 4 Discussion

Resistance to cell death, genome instability and mutation are the basic hallmarks of cancer (26). Interestingly, RCC cells were more sensitive to erastin-induced ferroptosis than others tumor cell types, which might be attributed to the dependence of GSH content and GPX4 activity to regulate redox homeostasis (18). Mechanistically, peroxisomes promoted the biosynthesis of polyunsaturated ether phospholipids (PUFA-ePLs), substrates of lipid peroxidation, and triggered the occurrence of ferroptosis. A decrease of PUFA-ePLs will promote the conversion of ferroptosis-sensitive state to ferroptosis-resistant state of RCC cells (27). Chemerin, a hypoxia-inducible factor (HIF)-dependent adipokine, suppressed fatty acid oxidation and thus mediated ferroptosis resistance in ccRCC (28). Moreover, one analysis revealed that ccRCC patients occurred a 2-82% mutation frequency among 36 ferroptosis-related genes (29). The multi-kinase inhibitors sorafenib is recommended to be the first-line strategy for treating advanced ccRCC patients (30, 31). Interestingly, it can block the system Xc^-^ function, induce GSH consumption and lipid ROS accumulation, and thus trigger ferroptosis in RCC cells (32–34). Therefore, comprehensive exploration of the FPTOSs expression profiles could deepen the understanding of occurrence and progression of ccRCC.

In the current study, using univariate Cox regression, LASSO regression, and multivariate Cox regression analyses, 5 FPTOSs with crucial prognostic significances were identified, including *ACADSB*, *CDCA3*, *CHAC1*, *MYCN*, and *TFAP2A*. Among them, *ACADSB* and *MYCN* were discovered as the protective factors, while *CDCA3*, *CHAC1* and *TFAP2A* were discovered as the detrimental factors. ACADSB is a member of acyl-CoA dehydrogenase family, and is predominantly involved in the processes of fatty acid metabolism, branch-chained amino acid metabolism and ferroptosis (35, 36). It was reported that *ACADSB* expression was positively associated with the expression of ferroptosis driving genes. Suppression of ACADSB was observed in ccRCC samples, which was accompanied with advanced grade and stage, and might function as an independent prognostic factor of ccRCC patients (37). CDCA3 engaged in cell cycle regulation through mediating ubiquitin degradation of mitosis-inhibitory kinase WEE1 (38). It was considered to be a prognostic factor of RCC, and the upregulation of *CDCA3* was associated with advanced TNM stage, tumor grade and immune cell infiltration (39). In addition, lncRNA SNHG12 increased *CDCA3* expression and thus mediated tumor progression and sunitinib resistance in RCC patients (40). CHAC1 was implicated in the processes of endoplasmic reticulum (ER) stress and ferroptosis (41). It could serve as a biomarker to independently forecast the prognostic outcomes of ccRCC patients, and was positively associated with the expression signatures of various immune cells (memory B cell, NK cell and Th1 cell) and ICI genes (*ADORA2A*, *CD200*, *CD44*) (42). Aberrant *MYCN* amplification was previously considered as a driving event of high-risk neuroblastoma (43). However, inhibition of MYCN contributed to the drug resistance of cisplatin through repressing apoptosis in epithelial ovarian cancer (44). The specific roles of MYCN in ccRCC progression still requires further verification. Transcriptional factor TFAP2A controlled the expression of various tumor-related genes including *VEGF*, *BCL-2*, *c-Kit* and *c-Myc*, and was reported to be widely upregulated in tumor samples (45). Additionally, suppression of TFAP2A inhibited cell proliferation, migration and invasion *via* initiating oxidative stress and ferroptosis in gallbladder carcinoma (46).

These 5 FPTOS genes were then included into a prognostic model, which was utilized to develop a risk scoring system, named FPTOS_score. All patients were allocated into low- and high-risk groups on the basis of the median value of FPTOS_score. The results indicated a poor prognosis existed in the high-risk group, and the prognostic model presented preferable predictive sensitivity and accuracy. What else, the FPTOS-based risk stratification was able to distinguish patients with undesirable outcomes, and the results were robust even after considering the influence of various clinical parameters.

miRNAs served as a class of crucial molecules that regulate gene expression in a post-transcriptional modification manner. It was reported that miRNAs were responsible for regulating ROS generation and thus promoting ferroptosis occurrence in ccRCC (47). Hence, we carried out a co-expression analysis to explore the crosstalk between differentially expressed miRNAs and prognostic FPTOSs, and a total of 30 miRNA-FPTOS regulatory pairs were obtained, which might bring novel insights into the gene regulation patterns in ccRCC.

Emerging evidences demonstrated that accumulation of somatic mutation events is responsible for the tumorigenesis and progression (48). TMB is newly considered as a substitute for neoantigen load to act as a prognostic biomarker for cancer (49). Therefore, identification of mutated genes especially driver genes of ccRCC may provide promising opportunities for personalized therapy and prognosis prediction. The findings indicated that patients from high-risk group performed elevated TMB level, which was accompanied with a poor prognosis. Abundance mutation events were existed in patients with high-risk, and the well-defined driver genes *VHL*, *PRBM1* and *TTN* occupied the most frequent mutation sites in both the low- and high-risk groups. Interestingly, patients from the high-risk groups experienced a worse prognosis than those from the low-risk groups when the mutation of these diver genes was taken into account. A recently accepted notion of RCC progression is that *VHL* mutation function as an initial event to drive tumorigenesis, while *PBRM1*, *BAP1* and *SETD2* subsequent trigger defects in DNA repair system and abnormal tumor growth (50). *TTN* mutation has been reported to be correlated with myopathy and cancer, and one study showed that lncRNA TTN-AS1, which is transcribed in the opposite direction of TTN, was upregulated in ccRCC samples and positive associated with poor clinicopathological performances (51).

The infiltration of immune cell was predicted using CIBERSORT algorithm. Herein, the tumor samples from high-risk group were infiltrated with CD8^+^ T cells, whereas those from low-risk group were infiltrated with resting mast cells. Unlike other solid tumors, there is a generally accepted viewpoint that increased CD8^+^ T cells infiltration in RCC samples was positively associated with weak outcome (52). This phenomenon might owe to a relative lack of tertiary lymphoid structures, which suppressed the mature process of dendritic cell, and thus prevented CD8^+^ T cells from recognizing tumor antigen (52, 53). Conversely, ccRCC tumor samples with abundant mast cell population performed better OS and PFS than those with scare mast cell population (54). Meanwhile, the immune score and estimate score were increased but the tumor purity was decreased in the high-risk group. The diversities of immune microenvironment might confer distinct drug susceptibilities to chemotherapy and immunotherapy. When compared with the low-risk group, the expression of ICI targeted genes (*PD-1, CTLA-4*) were significantly increased in the high-risk group. Patients with advanced or metastatic RCC have exhibited a desirable response rate to FDA-approved ICI drugs, such as anti-PD-1 antibody (nivolumab, pembrolizumab, atezolizumab) and/or anti-CTLA-4 antibody (ipilimumab) (55–58). Despite these advantages, most patients could not gain a durable response to immunotherapy. Encouragingly, the current study demonstrated that patients with high-risk performed a better response probability to anti-PD-1 immunotherapy than those with low-risk. Therefore, applying the FPTOS-based risk stratification might bring great benefits to metastatic RCC patients through distinguishing patients who respond positively to immunotherapy. Finally, correlation analysis indicated that cisplatin, BI-D1870 and docetaxel might serve as sensitive drugs to treat patients with high FPTOS_score.

Generally, the present study had mapped a ferroptosis and oxidative stress-associated landscape of ccRCC, and developed a prognostic model with a preferable predictive accuracy and stability. However, limitations should not be ignored. First, the transcriptome data were extracted from a retrospective cohort, and thus the prognostic model should be revaluated by a prospective cohort. Second, although robust results from bioinformatic analysis, the molecular functions and pathological mechanisms of the identified FPTOSs in ccRCC were still required experimental verification. Third, despite ICI-based immunotherapy and easily accessible drugs have shown the therapeutic potential for high-risk group, how to choose the optimum treatment protocol deserve further exploration.

## 5 Conclusion

Overall, we identified the FPTOSs with potential prognostic significance in ccRCC patients. A reliable score system to distinguish high-risk patients was established and performed a preferable predictive accuracy and stability. Subsequently, the miRNA-FPTOS regulatory network, driver gene mutation status, immune cell population, immunotherapy responsiveness, and drug susceptibility were examined. The results supply novel insights into the expression profiles of FPTOSs in ccRCC, and provide opportunities to identify therapeutical targets or prognostic biomarkers for ccRCC.

## Data availability statement

The clinical information of ccRCC patients is included in the supplementary material. The other original contributions presented in the study are publicly available. The data can be found here: TCGA database (https://portal.gdc.cancer.gov/) and ArrayExpress database (https://www.ebi.ac.uk/arrayexpress/). Further inquiries can be directed to the corresponding author.

## Author contributions

YW designed the study, performed the data analysis and interpretation. DL performed the data analysis and manuscript writing. BH performed the data collection. SZ revised the manuscript. All authors contributed to the article and approved the submitted version.
