# Supplementary material for: Exploring a ferroptosis and oxidative stress-based prognostic model for clear cell renal cell carcinoma
Source: Front Oncol. 2023 Mar 30;13:1131473. doi: 10.3389/fonc.2023.1131473 (PMC10098013; doi:10.3389/fonc.2023.1131473)
Supplement: Supplementary file 1 [file Image_1.pdf]

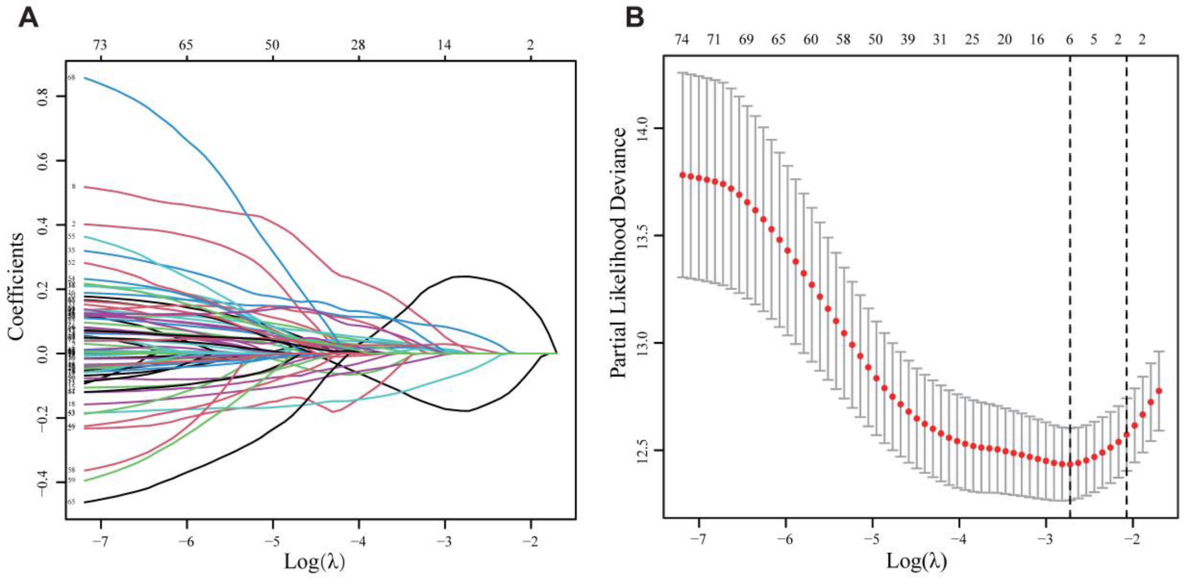

Supplemental Figure 1. Identifying crucial prognosis-related FPTOSs using LASSO regression analysis. **(A)** Trajectory variation of the independent variable coefficients. **(B)** The cross validation results of model construction.
