# Supplementary material for: Exploring a ferroptosis and oxidative stress-based prognostic model for clear cell renal cell carcinoma
Source: Front Oncol. 2023 Mar 30;13:1131473. doi: 10.3389/fonc.2023.1131473 (PMC10098013; doi:10.3389/fonc.2023.1131473)
Supplement: Supplementary file 2 [file Image_2.pdf]

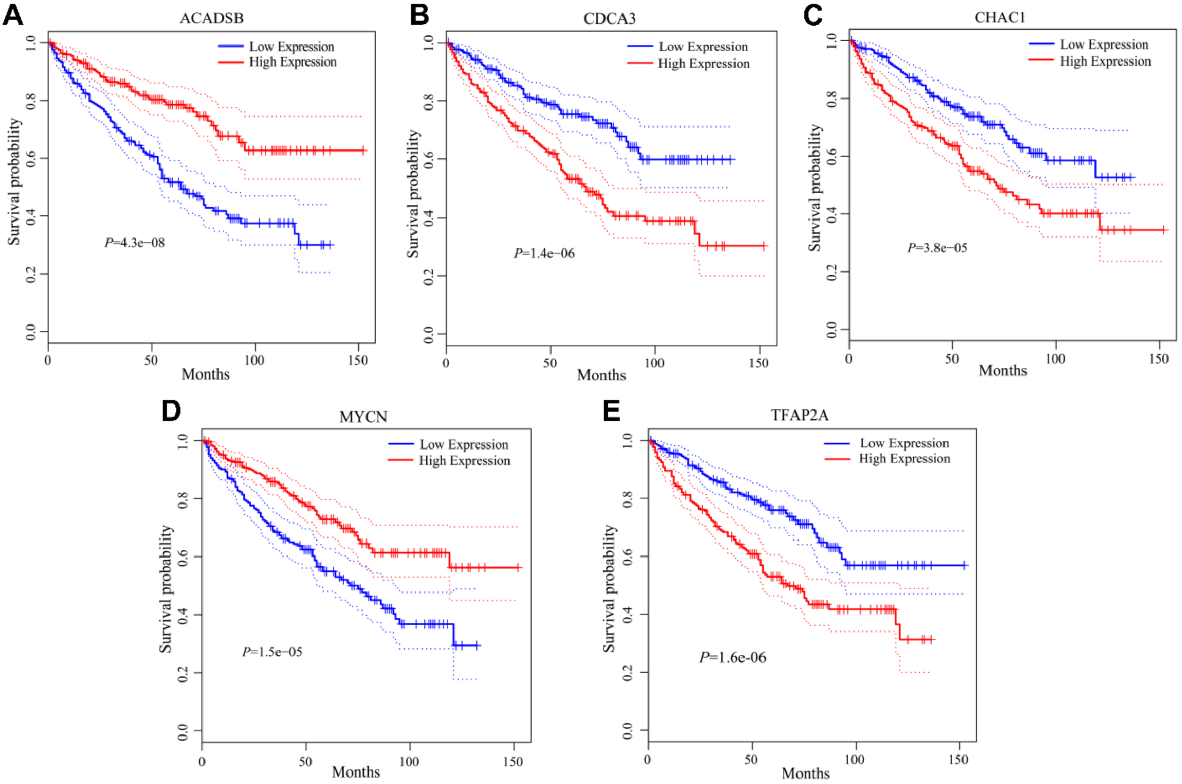

Supplemental Figure 2. Evaluating the prognostic values of the identified FPTOSs in ccRCC patients based on the GEPIA database. Kaplan-Meier survival analyses stratified by the relative expression of **(A)** *ACADSB*, **(B)** *CDCA3*, **(C)** *CHAC1*, **(D)** *MYCN*, **(E)** *TFAP2A*. The results indicated ACADSB and MYCN could serve as the favorable prognostic marker for ccRCC patients, while CDCA3, CHAC1, and TFAP2A could serve as the unfavorable prognostic marker for ccRCC patients. All data are available from the GEPIA database (http://gepia.cancer-pku.cn/index.html).
