# Supplementary material for: Exploring a ferroptosis and oxidative stress-based prognostic model for clear cell renal cell carcinoma
Source: Front Oncol. 2023 Mar 30;13:1131473. doi: 10.3389/fonc.2023.1131473 (PMC10098013; doi:10.3389/fonc.2023.1131473)
Supplement: Supplementary file 3 [file Image_3.pdf]

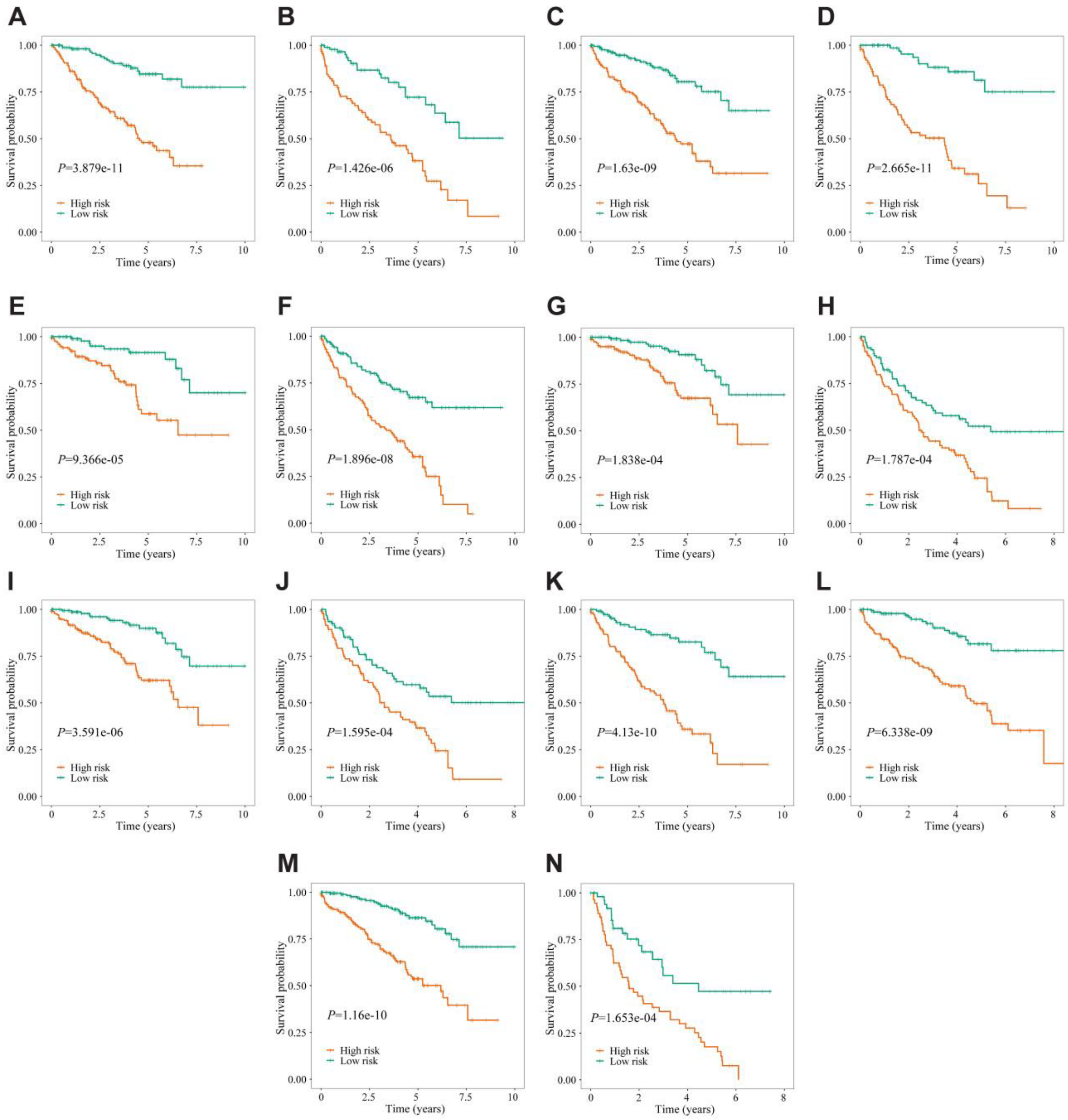

Supplemental Figure 3. Prognostic value of the FPTOS gene signature stratified by various clinical parameters. **(A)** age ≤ 65, **(B)** age＞65, **(C)** male, **(D)** female, **(E)** grade 1-2, **(F)** grade 3-4, **(G)** stage I-II, **(H)** stage III-IV, **(I)** T1-2, **(J)** T3-4, **(K)** N0, **(L)** N1-x, **(M)** M0, **(N)** M1-x. Log-rank test was applied to compare the statistical differences.
